# Photoelectrochemical device based on Mo-doped BiVO_4_ enables smart analysis of the global antioxidant capacity in food†
†Electronic supplementary information (ESI) available: Detailed materials and methods; the plot of the transformed Kubelka–Munk function *versus* the energy of light; EIS plots in the dark; XPS spectra; potential optimization; photocurrent response reproducibility in the PEC cell; fluorescence emission spectra; Mott–Schottky plot; CVs of antioxidants; linear equations, linear ranges and correlation coefficients for the antioxidants; different species of processed food analyzed; inner structure of the integrated device, *etc*. See DOI: 10.1039/c5sc02277k


**DOI:** 10.1039/c5sc02277k

**Published:** 2015-08-17

**Authors:** Lingnan Wang, Dongxue Han, Shuang Ni, Weiguang Ma, Wei Wang, Li Niu

**Affiliations:** a State Key Laboratory of Electroanalytical Chemistry , c/o Engineering Laboratory for Modern Analytical Techniques , Changchun Institute of Applied Chemistry , Changchun , Jilin 130022 , P. R. China . Email: dxhan@ciac.ac.cn ; Fax: +86-431-85262800 ; Tel: +86-431-85262425; b University of Chinese Academy of Sciences , Beijing 100039 , P. R. China; c Shenyang Agricultural University , Shenyang 110161 , P. R. China

## Abstract

For a healthy diet, which is an extension of a high quality lifestyle, tremendous attention has been focused on using antioxidant capacity indicators for food inspections and health guides. Although photoelectrochemical transducers have broadened our horizons for global antioxidant activity analysis, a growing body of foods and beverages needs to be quantified in the visible region and the necessary photoelectrochemical instrumentalization is still in its infancy. Generally, BiVO_4_ is considered as an ideal starting material for antioxidant surveillance under visible light irradiation. However, it is subjected to unsatisfied charge collection and utilization in practical applications. Herein, we studied the effects of successive molybdenum substitution of vanadium on the photocatalytic behavior of BiMo_*x*_V_(1–*x*)_O_4_ under visible light illumination. A superior photocurrent density was obtained for BiMo_0.015_V_0.985_O_4_ due to the flower-like architecture and favorable crystalline form. At the same time, this superhybrid BiMo_0.015_V_0.985_O_4_ composite successfully acted as a sensing unit in a photoelectrochemical platform for antioxidant capacity evaluation in foodstuffs. The related mechanism was further unearthed and discussed in-depth. Such a straightforward yet cogent principle was also applied to our integrated device for the “smart” analysis of the global antioxidant capacity, whereby collected data can be treated as a nutritive value index for routine quality control in the food industry. On the basis of this achievement, it is anticipated that mobile app-based quantitative antioxidant capacity detection will soon be realized.

## Abstract 2

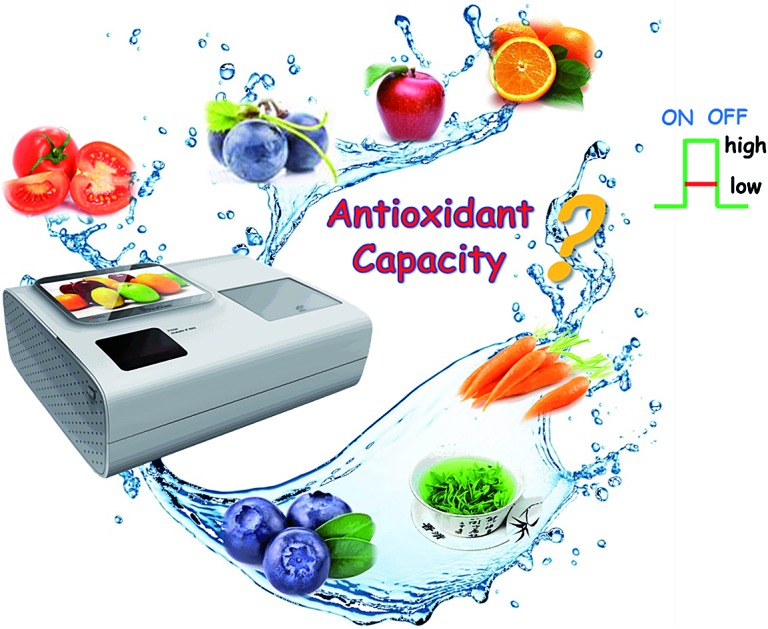
An ultrasensitive Mo-doped BiVO_4_ composite was used to engineer a photoelectrochemical platform for the direct analysis of the global antioxidant capacity. Using this principle, an integrated device was successfully exploited for the “smart” monitoring of antioxidant-rich foodstuffs.

## Introduction

Drawing inspiration from a healthy diet, the antioxidant capacity (AC) based on antioxidative substances in fresh fruits and vegetables has spurred intense interest and has evolved as a reliable index that should be a criterion for the comparison and classification of foodstuffs, even providing quality standards for regulatory issues and health claims.^1^ Recently, antioxidant nutrient content claims have been established as guidance for industry in food labeling by the Food and Drug Administration (FDA, USA), which indicates that food AC analysis has received comprehensive attention. Among the well-established methods, the photoelectrochemical (PEC) technique is considered to be an ideal platform for global AC assessment owing to its instinctive mechanism, portability, high sensitivity and prominent anti-interference.^2^ To deliver a sophisticated PEC system, eminent photoactive materials ought to possess appropriate band edge positions, band gap energies and a good photostability.^3^ In particular, the conduction and valence bands of preferential semiconductors should embrace the redox potentials of the overall natural antioxidants (AOs), which results in the possibility to make photo-induced holes react with all the AOs. For multitudinous semiconductors, n-type metal oxides have become the most sought-after candidates for food inspections, since they have moderate costs, possess highly reactive holes and are abundant, eco-friendly, and corrosion resistant. Bearing these properties in mind, advanced TiO_2_-based nanomaterials appear to be the benchmark material for dietary evaluation.^4^ However, the broad band gap in bare TiO_2_ results in a limited PEC performance.^5^ To our knowledge, shifting the optical absorption threshold into the visible region would not only result in a breakthrough in the photoresponse, but also cut the cost for apparatus construction.

Ternary metal oxide BiVO_4_ is deemed to be a valid alternative semiconductor for visible light harvesting.^6^ It is worth mentioning that the morphology and microstructure profoundly impact on the properties of BiVO_4_.^7^ There are three typically known polymorphs of BiVO_4_ found in nature, monoclinic scheelite, tetragonal scheelite and tetragonal zircon, with the monoclinic scheelite structure exhibiting the best photocatalytic activity.^8^ Unfortunately, the actual photocatalytic behavior achieved with pristine BiVO_4_ is far below that expected, which is due to its inferior charge–carrier separation and poor surface absorption.^9^ To circumvent these problems, impurity doping of metallic and nonmetallic elements into BiVO_4_ seems to be a hot strategy, which can regulate the composition to further modify its optoelectronic efficiency. For instance, using molybdenum to replace the partial sites of vanadium was found to enhance the electronic conductivity and ultimately reduce the likelihood of electron–hole recombination in the bulk.^10^ Quite interestingly, VO_4_ units in the scheelite-type BiVO_4_ are isolated, which drives the photogenerated electrons to hop between V neighbors since the conduction band (CB) has a primary contribution from the V 3d orbitals.^11^ Displacing vanadium with high-oxidation state molybdenum causes crystal deformation and polaron transport, thus upscaling the charge carrier extraction efficiency.^12^ Nevertheless, dopants can also be a double-edged sword, either exhibiting a superior photocurrent density or tipping the balance as scattering centers when the recommended ratio is exceeded.^13^ Meanwhile, differences in the transport properties cannot rely solely on heteroatoms; special surface features from diverse fabrication routes play a key role in compensating for self-trapping.^14^ Until now, the close relationship between the structure transformation and the variation in PEC properties has not been thoroughly discussed.

**Fig. 1:**
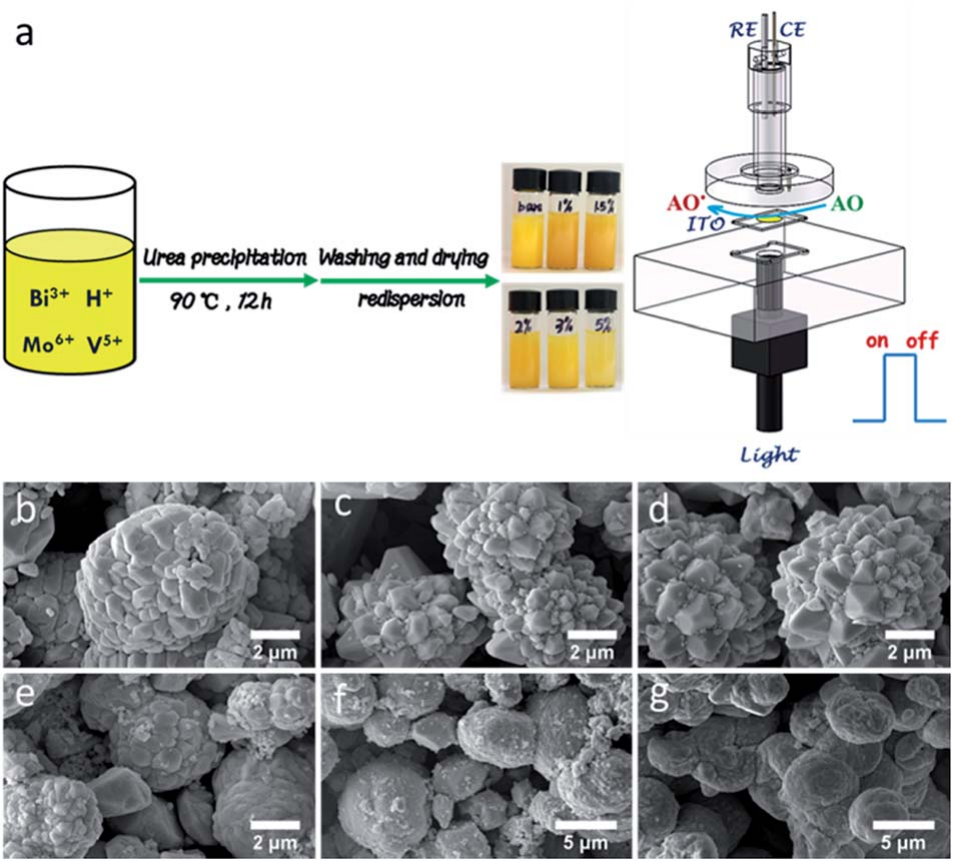
(a) Schematic illustration for the preparation of BiMo_*x*_V_(1–*x*)_O_4_ and the PEC process for the AC assay. FESEM images of *x* = (b) 0, (c) 0.01, (d) 0.015, (e) 0.02, (f) 0.03 and (g) 0.05 for BiMo_*x*_V_(1–*x*)_O_4_.

Here, we report a one-pot chemical synthesis of BiMo_*x*_V_(1–*x*)_O_4_, where substituting lattice V with Mo brought distinct changes in the crystal structure and optical absorption properties. In depth, the corresponding interrelationship was adequately explored, giving a better understanding of doped BiVO_4_ systems. With appropriate doping, an exquisite PEC platform has been designed for rationally estimating the AC (Fig. 1a). Using this framework, an integrated device for the smart monitoring of the AC in food has been successfully developed. This portable, easy-to-use and cost effective bioassay equipment offers unrivalled convenience for consumers, allowing them to maintain a well balanced nutritional diet.

## Results and discussion

Engineering the PEC platform mostly included two straightforward procedures: (1) synthesis of tunable Mo-doped BiVO_4_*via* a urea-precipitation reaction; (2) manipulating the optimal composition in terms of the property-to-performance trends (Fig. 1a). The series of field emission scanning electron microscopy (FESEM) images in Fig. 1b–g reveals the diverse morphology as the Mo content increases. It can be observed that bulk BiVO_4_ has loose microspheres and a smooth surface (Fig. 1b). When the Mo mole fraction is less than or equal to 1.5% [*i.e.* mol (Mo) per mol (Mo + V) = 0.015], a large number of protuberances were formed, resulting in a flower-like architecture (Fig. 1c and d). Concomitant improvements in the surface active sites may breed on the localized gibbous topographies. Instead, higher doped samples (>1.5%) suffered serious aggregation, which dramatically decreased its surface-to-volume properties (Fig. 1e–g) as well as its water solubility (Fig. 1a).

**Fig. 2:**
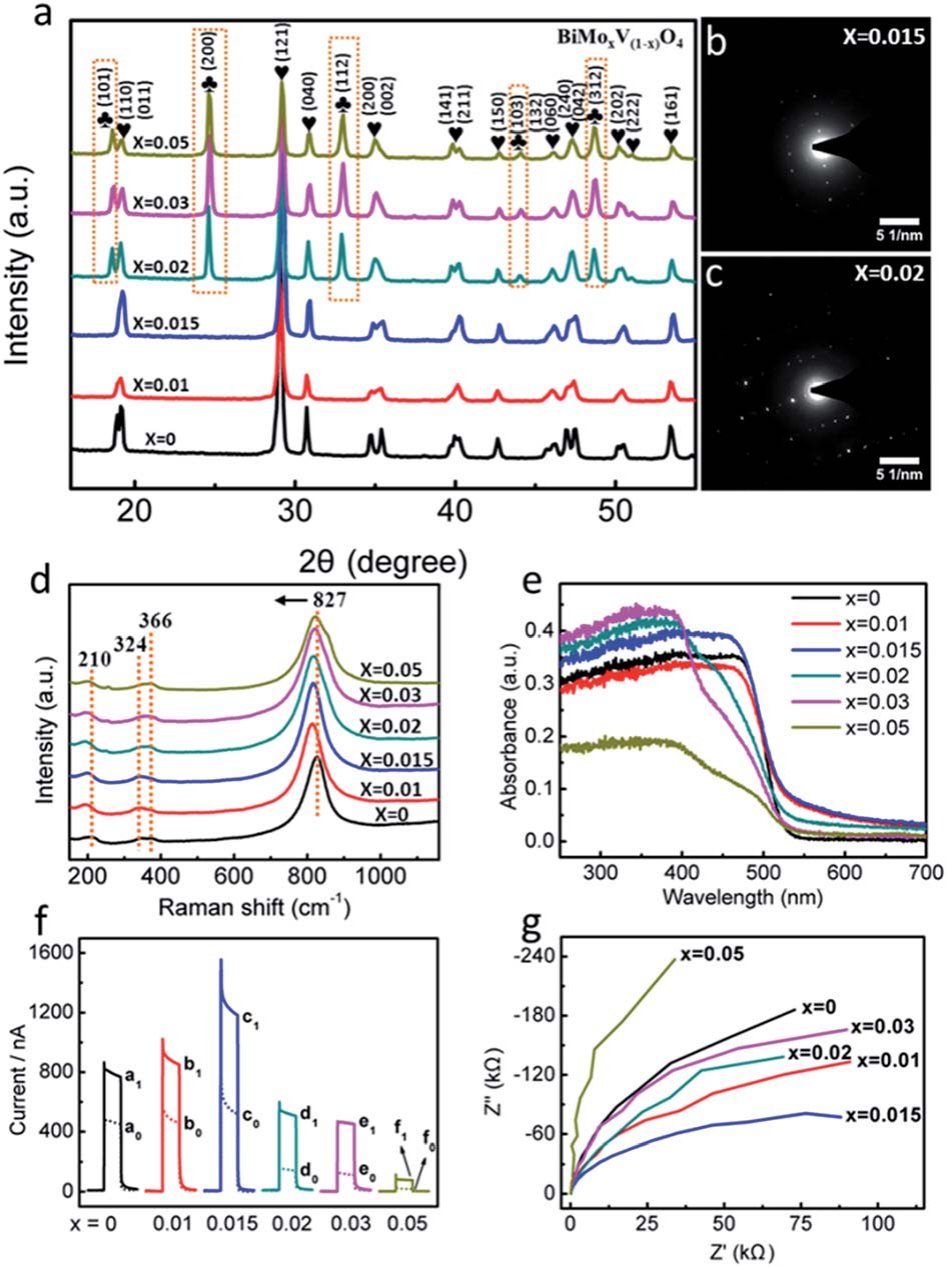
(a) XRD patterns of BiMo_*x*_V_(1–*x*)_O_4_. SAED patterns of *x* = (b) 0.015 and (c) 0.02. (d) Raman spectra and (e) UV-vis DRS of BiMo_*x*_V_(1–*x*)_O_4_. (f) Photocurrent responses of the BiMo_*x*_V_(1–*x*)_O_4_ modified ITO electrode in the absence of (dot curves a_0_–f_0_) and presence of (solid curves a_1_–f_1_) 74.44 μmol L^–1^ gallic acid. The PEC sensors were applied at 0 V under 470 nm light excitation in 0.1 mol L^–1^ PBS (pH = 7.4). (g) EIS plots measured at 0.3 V (*vs.* Ag/AgCl) in 1 mol L^–1^ Na_2_SO_4_ solution under 470 nm light for BiMo_*x*_V_(1–*x*)_O_4_.

X-ray diffraction (XRD) analysis was conducted to investigate the phase structure of BiMo_*x*_V_(1–*x*)_O_4_ (Fig. 2a). Clearly, the diffraction patterns of the samples with a low dopant concentration (*x* = 0.01 and 0.015) are nearly identical to that of pristine BiVO_4_, which is in perfect agreement with the monoclinic scheelite structure (JCPDS 014-0688) without any impurity phases.9*a* However, with increasing Mo amounts (*x* = 0.02, 0.03 and 0.05), characteristic peaks of the tetragonal structure (JCPDS 014-0133) at 24.6°, 32.9°, 44.0° and 48.6° were observed,^15^ indicating that the crystal symmetry continuously changes from monoclinic to tetragonal. The crystal deformation may be ascribed to substitutional defects of Mo^6+^ ions *in lieu* of V^5+^, since the tetrahedral ion radius of Mo^6+^ is slightly greater than that of V^5+^. As a matter of fact, with the introduction of Mo, the typical crystal planes (121) and (040) in all samples gradually shifted to smaller angles.^16^ The XRD results also confirm that Mo^6+^ has been embedded into the V^5+^ sites of the host BiVO_4_ lattice, expediting lattice expansion. The selected area electron diffraction (SAED) patterns in Fig. 2b and c show a distinct crystal transition tendency. Excess impurities make the single crystal diffraction images intricate, which match well with the outcomes of the XRD spectra.

To probe the building composition and local distortions of BiMo_*x*_V_(1–*x*)_O_4_, Raman spectra were measured (Fig. 2d). Raman bands at approximately 210, 324, 366, and 827 cm^–1^ were observed for all samples. The band centered at 210 cm^–1^ gives little structural information, it just represents the external mode of BiVO_4_.^17^ The two bands at 324 and 366 cm^–1^ are in accordance with the asymmetric–symmetric bending vibrations of the VO_4_^3–^ tetrahedron.^18^ The dominant band at 827 cm^–1^, assigned to the stretching mode of V–O bonds, began to shift negatively as Mo became involved, which implies that the local structure change is a driving force for the longer V–O bond length.^19^

For a better overview of the electronic structure in the BiMo_*x*_V_(1–*x*)_O_4_ nanomaterials, UV-visible diffuse reflectance spectra (DRS) were measured (Fig. 2e). When the ratio is set to 1.5% at the vanadium sites (BiMo_0.015_V_0.985_O_4_), the visible light absorption properties are optimal. A plot of the transformed Kubelka–Munk function *versus* the energy of light (Fig. S1, ESI†) roughly estimates the bandgap in six composites, and the narrowest band gap energy belongs to BiMo_0.015_V_0.985_O_4_.

In order to deeply understand the optoelectronic properties of BiMo_*x*_V_(1–*x*)_O_4_, photoelectronic response trials were performed and they are displayed in Fig. 2f. Bare BiVO_4_ showed a moderate photocurrent increment (defined as the PEC current, *I* = *I*_sample_ – *I*_blank_ = 318.2 nA) at a potential of 0 V with 470 nm illumination. After doping various amounts of Mo in, the PEC current soared by 1.23 (1.0%) and 2.09 times (1.5%) compared to pristine BiVO_4_. While the Mo mole fraction climbed to 2%, 3% and 5%, the PEC current quickly fell back. Unquestionably, BiMo_0.015_V_0.985_O_4_ demonstrates the highest photocatalytic behavior. A similar tendency is also echoed by the electrochemical impedance spectroscopy (EIS) plots under visible light (Fig. 2g), where BiMo_0.015_V_0.985_O_4_ presents the smallest arc radius, indicating the optimum conductivity.

Since BiMo_0.015_V_0.985_O_4_ manifests a preferable band gap, photocurrent response and electronic transport capability, it is wise to choose a Mo mole fraction of 1.5% as the optimal photosensitive constituent. The explanation might be speculated as follows. (1) With a doping ratio of less than 1.5%, V substitution by a small amount of Mo sustains a high level of ligand π-back bonding with a low d-electron count, which breaks the detrimental localization of the V d-orbitals due to the weak overlap with the Bi 6p orbitals.^20^ In addition, depending on the circumstances of the EIS measurements in the dark (Fig. S2, ESI†), the smaller resistance for BiMo_0.015_V_0.985_O_4_ implies an efficient improvement in the photocarrier transport and extraction *via* Mo doping. (2) As the Mo level surpasses 1.5%, the photocurrent response soon decreases, and becomes even worse than that of the original BiVO_4_. The overload of Mo turns into carrier recombination centers, which further impedes the charge transport. It should be asserted that both the larger surface reactive junction area and crystallinity are essential aspects for the PEC activity. Such a flower-like appearance and monoclinic scheelite structure in BiMo_0.015_V_0.985_O_4_ guarantee desirable characteristics for antioxidant detection.

**Fig. 3:**
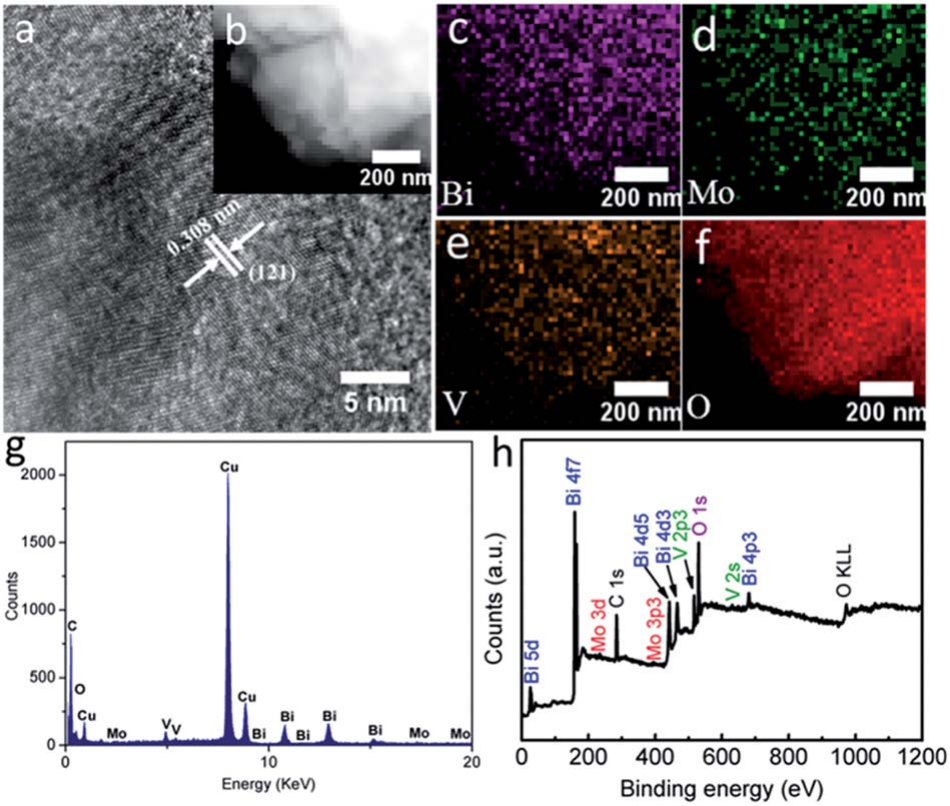
(a) The HETEM image, (b) HAADF-STEM image, elemental mapping images of (c) Bi, (d) Mo, (e) V and (f) O, (g) EDX spectrum and (h) the survey XPS spectrum of BiMo_0.015_V_0.985_O_4_.

Valuable insights into the intrinsic features of BiMo_0.015_V_0.985_O_4_ were then surveyed thoroughly by high-resolution transmission electron microscopy (HRTEM), element mapping, energy dispersive X-ray (EDX) spectroscopy and X-ray photoelectron spectroscopy (XPS). The interplanar spacing distance in the HRTEM image (Fig. 3a) reaffirms that the (121) lattice plane is present in the BiMo_0.015_V_0.985_O_4_ nanocrystal. Besides, high-angle annular dark-field scanning transmission electron microscopy (HAADF-STEM) and element mapping (Fig. 3b–f) show a merely homogeneous Bi, Mo, V and O element distribution, in accordance with the EDX (Fig. 3g) spectrum and the survey XPS (Fig. 3h). The EDX spectrum (Fig. 3g) was collected using a TECNAI G2 microscope operating at 200 kV. In fact, the C and Cu signals arise from the carbon-film-coated copper grid. The sample that we used to gather the data was close to the edge of the copper grid. Therefore, the intensity of the impurity peaks is strong. Moreover, the chemical states of the BiMo_0.015_V_0.985_O_4_ surfaces are shown in Fig. S3a–c (ESI†). The Bi 4f peak is deconvoluted into two parts, at 159.0 and 164.3 eV, corresponding to Bi 4f_2/7_ and Bi 4f_2/5_, respectively.^21^ The split peaks of Mo 3d at 232.1 and 235.3 eV are fitted to Mo 3d_5/2_ and Mo 3d_3/2_, suggesting that the Mo cations mostly behave in the Mo^6+^ oxidation state in the V places in BiMo_0.015_V_0.985_O_4_.10*b* Fig. S3c (ESI†) shows the sole form of V^5+^ in VO_4_^3–^, on account of the doublet peaks located at 516.9 and 524.6 eV, which are separately in line with V 2p_3/2_ and V 2p_1/2_.^22^

**Fig. 4:**
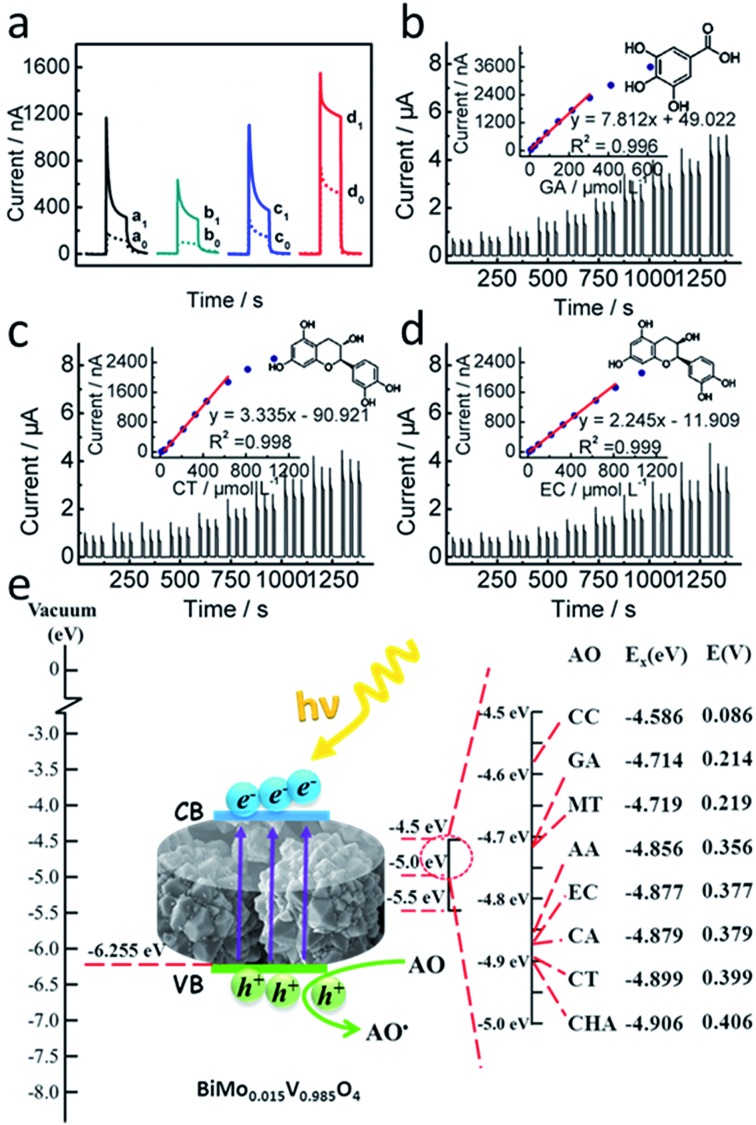
(a) Photocurrent responses of P25 (a_0_ and a_1_), utg-C_3_N_4_/TiO_2_ (b_0_ and b_1_), SGE–TiO_2_ (c_0_ and c_1_) and BiMo_0.015_V_0.985_O_4_ (d_0_ and d_1_) modified ITO electrodes without (dots) and with (solid curves) 74.44 μmol L^–1^ GA. Concentration-dependent photocurrent of different AOs (GA (b), CT (c) and EC (d)). The insets in (b), (c) and (d) are the linear curves of GA, CT and EC, respectively. The PEC sensors were applied at 0 V under 470 nm light excitation in 0.1 mol L^–1^ PBS (pH = 7.4). (e) The mechanism of the PEC sensor for the AC evaluation. AO: antioxidant; *E*_x_ (eV): the redox potential of the AOs with respect to a vacuum; *E* (V): the redox potential of the AOs (*vs.* NHE).

PEC technology has received flourishing research interest as a result of creatively inheriting from both optical and electrochemical methods.^23^ The selection of photoactive volunteers is of paramount importance to achieve superiority during visible photocatalysis. The contrast test shown in Fig. 4a sheds new light on the favorable visible-light-responsive properties of BiMo_0.015_V_0.985_O_4_. It should be noted that ultrathin graphitic carbon nitride/titanium dioxide (utg-C_3_N_4_/TiO_2_) and sulfonated graphene–TiO_2_ (SGE–TiO_2_) were previous reported by our lab.^2^,^24^ Conspicuously, commercial titanium dioxide P25 exhibited a feeble photocurrent response (111.4 nA, curve a_0_) under visible light irradiation. Similarly, even if utg-C_3_N_4_ or SGE was utilized to modify TiO_2_ (curves b_0_ and c_0_), there were no real differences under 470 nm light. To our surprise, compared with TiO_2_-based nanohybrids, BiMo_0.015_V_0.985_O_4_ was easier to excite, which led to a fairly strong signal (518.6 nA, curve d_0_). Moreover, in the presence of 74.44 μmol L^–1^ gallic acid (GA), the photocurrent intensity of BiMo_0.015_V_0.985_O_4_ greatly increased to 1183 nA (d_1_), reaching 3.75, 3.89 and 3.11 times that of P25 (a_1_), utg-C_3_N_4_/TiO_2_ (b_1_) and SGE-TiO_2_ (c_1_), respectively. It has to be noted that BiMo_0.015_V_0.985_O_4_ is capable of shifting the threshold of the photoresponse into the visible range effectively, therefore providing a feasible way for developing low-cost antioxidant assay instruments in the near future.

For the sake of AC evaluation, a novel PEC cell has been designed (Fig. 1a). This device can realize a prompt signal acquisition for multiple AC assessments. During the examination, 0 V was chosen for the working potential in consideration of the advisable sensitivity and convenience for PEC device integration (Fig. S4, ESI†). Under the optimum conditions, AC analysis of some standard AOs commonly found in food were accomplished, including GA, (–)-epicatechin (EC), chlorogenic acid (CHA), (+)-catechin hydrate (CT), caffeic acid (CA), ascorbic acid (AA), cyanidin chloride (CC) and myricetin (MT). As listed in Table S1 (ESI†), all of the eight typical AOs expressed remarkable responses together with a wide linear range. Taking GA, CT and EC for instance, upon successive addition, the PEC current presented a linear increase and then approached a plateau (Fig. 4b–d). In light of a timely response for the main AOs in food, the global AC could be expressed as GA equivalents. Most excitingly, this conceptual strategy competently assesses the comprehensive antioxidant status rather than the individual AO concentration in food, which would allow customers to view the AC holistically. It is noticeable that after 33 repeated on–off detections, upon the addition of 74.44 μmol L^–1^ GA, the sensor still showed 93.58% of the initial signal, which proves a satisfactory reproducibility (Fig. S5, ESI†). Five electrodes prepared independently were used for the 74.44 μmol L^–1^ GA assay, and its relative standard deviation (RSD) was 2.95%. Given this fact, when three electrodes were stored in the dark, at least 95.50% of the primitive PEC current could be picked up a month later.

Referring to correlative literature, the major AC assays can be summarily divided into two categories: (1) hydrogen atom transfer (HAT) reactions, and (2) electron transfer (ET) reactions.^25^ As depicted in Fig. 4e, the valence band (VB) of BiMo_0.015_V_0.985_O_4_ was calculated to be –6.255 eV by virtue of the Mott–Scottky plot (Fig. S7, ESI†) and the plot of the transformed Kubelka–Munk function *versus* the energy (Fig. S1, ESI†), which is likely to oxidize water molecules to generate hydroxyl radicals. Once radicals accumulate, terephthalic acid (TA) can scavenge them with the relevant phenomenon reflected in the fluorescence spectrum.^26^ What is surprising is that the fluorescence peak situated at 425 nm (Fig. S6, ESI†) hardly changed as time went on. Hence, this system should be classified as an ET process on the basis of the direct reaction between the AOs and trapped holes. An in-depth perspective into the PEC mechanism is postulated as follows. Apart from the fact that the inert lattice mismatching in BiMo_0.015_V_0.985_O_4_ may compensate for the slow carrier mobility, Mo serves as shallow donors to make excess electrons form small polarons around reduced V^4+^ centers.^27^ A multitude of polarons help charge migrate to an adjacent V^5+^ site and render potent overlap on a large scale. Undoubtedly, it weakens the hopping activation energy for unconnected V neighbors, thereby minimizing the inner resistance and facilitating electron transport. In this sense, composition regulation and morphology innovation endow BiMo_0.015_V_0.985_O_4_ with a suitable band alignment to easily absorb visible light. Thereupon, electrons excited in the VB will transfer to the CB, leaving holes behind. The high-energy electrons promptly arrive at the ITO surface, which eventually gives rise to a remarkable photocurrent output. As expressed in Fig. S8 (ESI†), the CB and VB locations of BiMo_0.015_V_0.985_O_4_ bestride the formal potentials of the eight AOs. Aside from the electrolyte, if AOs exist in the solution, positive charge holes are likely to be replenished by electrons from the AOs and quickly get ready for the next photoexcitation. Due to this unique regenerative ability, the amplified signal can be utilized for overall AC assessment.

**Fig. 5:**
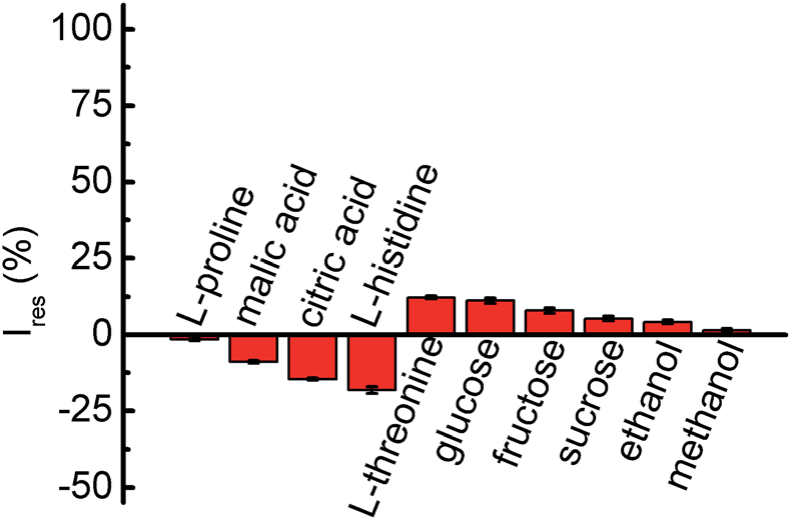
Photocurrent responses of the BiMo_0.015_V_0.985_O_4_ modified ITO electrode upon the addition of 10 mmol L^–1^ of l-malic acid, 20 mmol L^–1^ of l-citric acid, 25 mmol L^–1^ of l-threonine, 40 mmol L^–1^ of glucose or fructose, 50 mmol L^–1^ of l-histidine, or 250 mmol L^–1^ of l-proline, sucrose, ethanol or methanol in 0.1 mol L^–1^ PBS (pH = 7.4) containing 0.5 mmol L^–1^ GA at 0 V under 470 nm light excitation.

With respect to a complex food system, coexisting substances, such as amino acids, polysaccharoses and organic acids, are always present. Attributing to an actual situation, a tentative trial was carried out, which contained 0.5 mmol L^–1^ GA accompanied by some interfering components (Fig. 5). Evidently, an insignificant photocurrent decay was seen when 250 mmol L^–1^ of l-proline, sucrose, ethanol or methanol, 50 mmol L^–1^ of l-histidine, 40 mmol L^–1^ of glucose or fructose, 25 mmol L^–1^ of l-threonine, 20 mmol L^–1^ of l-citric acid, or 10 mmol L^–1^ of l-malic acid were involved. On this point, the PEC transducer shows universality for practical applications.

The feasibility of rational AC estimation was further pursued using different foodstuffs. Eight kinds of fresh fruit, as natural food, was firstly explored (Table 1). For these samples, the juice extracts were gathered and centrifuged before detection. Besides natural food, this PEC strategy is also suitable for processed foods, such as tea, wine, coffee, drinks, *etc.* In this study, some different commercial teas and beverages were analysed as examples (Table 2, Table S2, ESI†). To investigate the reliability of the PEC platform, Folin–Ciocalteu (F–C) and 2,2-diphenyl-1-picryhydrazyl radical (DPPH) approaches, as experienced optical methods, were simultaneously carried out. As is well known, the outcome differences between PEC and DPPH methods should be due to the different mechanisms and calibration standards. Likewise, although PEC and F–C measurements are both grouped into the ET reaction group and take GA as an equivalent, the final test results of the PEC assay often present relatively small values. For colorimetric total phenolic analysis, exterior colors, reducing agents and possibly metal chelators can interfere with the F–C method. As for dark-colored systems, F–C detection might make a big sacrifice in the precision. In contrast, the PEC sensor adopts back-side illumination to avoid any background color effect, thereby attaining a more reasonable accuracy. Despite all of this, it can be seen in Tables 1 and 2 that the data from PEC, F–C and DPPH were exactly consistent with each other. Due to the superior high sensitivity, convenient operability and outstanding anti-interference properties, the PEC platform is thus efficient for the evaluation of antioxidants in food.

**Table 1:** The antioxidant capacities of fresh fruits as found with our PEC sensor, the F–C method and the DPPH method. Data are expressed in mg L^–1^ for the fruits (*n* = 3)

Practical samples	PEC sensor	F–C method	DPPH method
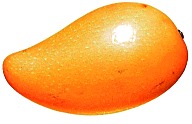	356.38 ± 6.80	369.73 ± 2.63	267.61 ± 1.46
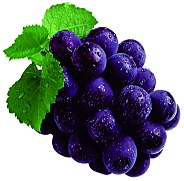	229.40 ± 1.13	247.71 ± 0.61	172.73 ± 2.92
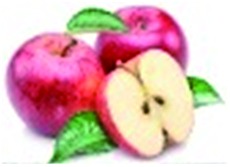	257.45 ± 4.95	252.01 ± 2.43	176.86 ± 2.53
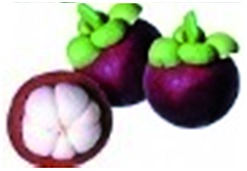	265.54 ± 6.63	308.44 ± 5.27	182.01 ± 3.86
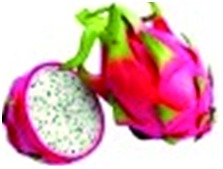	104.18 ± 3.14	129.27 ± 0.38	83.76 ± 0.73
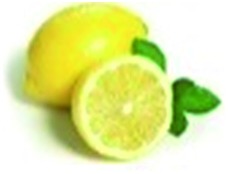	445.59 ± 9.12	468.67 ± 1.52	281.66 ± 1.82
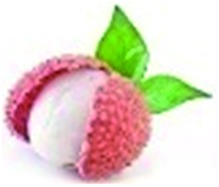	540.48 ± 1.01	564.39 ± 3.04	325.50 ± 3.16
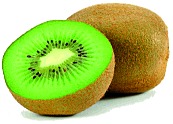	601.12 ± 5.38	611.73 ± 4.56	344.83 ± 11.38

**Table 2:** The antioxidant capacities of commercial teas (T) and drinks (D) as found with our PEC sensor, the F–C method and the DPPH method. Data are expressed in mg g^–1^ for the teas and in mg L^–1^ for the drinks (*n* = 3)

Practical samples	PEC sensor	F–C method	DPPH method
T1	18.78 ± 0.80	35.82 ± 0.25	54.84 ± 0.24
T2	73.33 ± 1.72	106.56 ± 0.53	144.03 ± 0.42
T3	108.63 ± 1.91	120.87 ± 0.70	161.18 ± 0.85
T4	52.11 ± 1.51	71.60 ± 0.23	106.90 ± 0.73
D1	387.19 ± 0.62	420.30 ± 1.07	203.64 ± 2.39
D2	345.31 ± 1.24	386.24 ± 6.77	193.31 ± 2.30
D3	396.16 ± 4.34	437.52 ± 1.32	318.36 ± 7.59
D4	429.88 ± 4.24	459.78 ± 2.97	325.78 ± 2.39
D5	316.78 ± 0.98	337.46 ± 2.21	185.21 ± 1.53
D6	393.10 ± 0.98	435.85 ± 1.14	250.63 ± 4.08
D7	457.50 ± 3.01	492.08 ± 1.14	566.88 ± 3.28
D8	712.10 ± 13.04	770.78 ± 1.02	731.51 ± 1.55
D9	641.11 ± 9.59	701.33 ± 3.70	728.08 ± 1.37
D10	436.60 ± 4.37	487.50 ± 0.41	522.16 ± 4.07
D11	296.41 ± 0.76	310.27 ± 1.70	164.06 ± 2.06
D12	352.02 ± 5.05	388.18 ± 0.62	198.06 ± 2.06
D13	391.22 ± 1.42	432.17 ± 1.03	232.09 ± 4.07
D14	176.01 ± 0.76	137.02 ± 0.52	45.29 ± 1.63
D15	58.07 ± 0.36	34.43 ± 0.19	27.96 ± 0.73
D16	59.29 ± 0.27	40.32 ± 0.18	31.07 ± 0.48

**Fig. 6:**
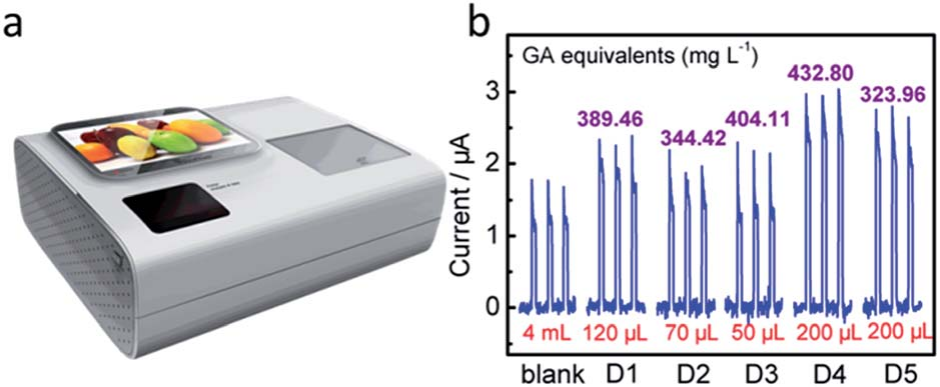
(a) Photograph of the integrated PEC platform. (b) Photocurrent responses of the BiMo_0.015_V_0.985_O_4_ modified ITO electrode in the integrated PEC platform without (blank PBS solution) and with the addition of a certain volume of juice to 4 mL of 0.1 mol L^–1^ PBS (pH = 7.4).

To date, research and development of commercial PEC apparatus for AO analysis are in their infancy. After unremitting efforts, we have accomplished the development of an integrated home-made device embedded with a testing system, data collection unit, and processing and information output units (Fig. 6a and Fig. S9, ESI†). The internal flow-cell system obtained a linear regression equation of GA, namely, *y* (μA) = 0.01056*C* (μmol L^–1^) – 0.09377 (*R*^2^ = 0.998). When five of the above-mentioned commercial drinks were examined, each of the photocurrent responses from the home-made device was found to show good consistency with the calculated values of PEC sensors (Fig. 6b, Table 2). Of course, the embedded equipment is highly robust and automatic, and meets the requirements for “smart” AC assessment for layfolk.

## Conclusions

In summary, diminutive lattice and morphology variations in Mo-doped BiVO_4_ have a significant effect on the photoactivity. With the aid of adjustable impurity doping, flower-like BiMo_0.015_V_0.985_O_4_ explicitly exerts great advantages for the prompt communication with AOs. The ultrasensitive BiMo_0.015_V_0.985_O_4_ based PEC platform has blossomed into a versatile format to estimate the AC in food. Based on this principle, an engineered fluidic device was exploited for real-time measurements in antioxidant food samples against a set of controls. The integrated device shows a rapid response, long-lasting stability, anti-interference properties and universality for “smart” AC analysis, meeting the approval of personnel without highly trained experience. This proof-of-concept is seen as a giant step for progressively intelligent mobile detection of AC in the foodstuff industry, even opening up a bright future for cosmetic and healthcare supervision.

## Supplementary Material

Supplementary information
