# Supplementary material for: Photoelectrochemical device based on Mo-doped BiVO4 enables smart analysis of the global antioxidant capacity in food
Source: Chem Sci. 2015 Aug 17;6(11):6632–8. doi: 10.1039/c5sc02277k (PMC5802276; doi:10.1039/c5sc02277k)
Supplement: Supplementary file 1 [file SC-006-C5SC02277K-s001.pdf]

Electronic Supplementary Material (ESI) for Chemical Science.
This journal is © The Royal Society of Chemistry 2015

Photoelectrochemical Device Based upon Mo-doped $BiVO_4$ Enable Smart Analysis of Global Antioxidant Capacity in Food**

Lingnan Wang,[ab] Dongxue Han,[*a] Shuang Ni,[ac] Weiguang Ma,[ab] Wei Wang[a] and Li Niu[a]

[a] State Key Laboratory of Electroanalytical Chemistry, c/o Engineering Laboratory for Modern Analytical Techniques, Changchun Institute of Applied Chemistry, Changchun, Jilin 130022, P. R. China.

[b] University of Chinese Academy of Sciences, Beijing 100039, P. R. China

[c] Shenyang Agricultural University, Shenyang 110161, P. R. China

1. Experimental section

1.1 Materials

Bismuth (III) nitrate pentahydrate ($Bi(NO_3)_3{\cdot}5H_2O$), ammonium vanadium oxide (NH_4VO_3), urea, ammonium molybdate (para) tetrahydrate ($(NH_4)_6Mo_7O_{24}{\cdot}4H_2O$) and gallic acid (GA) were purchased from Alfa (China). 2,2-diphenyl-1-picryhydrazyl radical (DPPH) was obtained from Alfa (USA). Terephthalic acid (TA) was bought from Alfa (UK). Glucose, (+)-catechin hydrate (CT) and caffeic acid (CA) were purchased from Sigma-Aldrich (China). Ascorbic acid (AA) was obtained from Sigma-Aldrich (UK). 6-hydroxy-2,5,7,8-tetramethylchromane-2-carboxylic acid (Trolox) and Folin-Ciocalteu (F-C) reagent (10 %) were purchased from Sigma-Aldrich (Switzerland). (-)-Epicatechin (EC) was bought from Aladdin (China). Chlorogenic acid (CHA) was got from J&K Chemical (China). Myricetin (MT) was obtained from TCI (Japan). Cyanidin chloride (CC) was purchased from Sinopharm Chemical Reagent Co,Ltd (China). All reagents were used as received without further purification. The PBS buffer was made from sodium phosphate (NaH_2PO_4/Na_2HPO_4, 81:19 (molar ratio)) and sodium chloride, which were dissolved in deionized water at final concentrations of 10mmol L^{-1} (pH=7.4).

1.2 Sample Preparation

Preparation of $BiVO_4$. Pure $BiVO_4$ was prepared according to the urea-precipitation method. In a typical procedure, 5.821 g $Bi(NO_3)_3{\cdot}5H_2O$ and 1.404 g NH_4VO_3 were separately dissolved in 50 mL nitric acid (2 mol L^{-1}). After mixing these two solutions and continuous stirring for 30 min, a stable clear solution was obtained. Subsequently, 5 g urea was added into the mixture and heated upto 90 °C for 24 h with constant stirring. The resultant product was rinsed thoroughly with water and dried at 100 °C for 12 h.

Preparation of Mo-doped $BiVO_4$. The preparation of Mo-doped $BiVO_4$ samples are similar to that of pure $BiVO_4$. Specifically, $Bi(NO_3)_3{\cdot}5H_2O$, NH_4VO_3 and $(NH_4)_6Mo_7O_{24}{\cdot}4H_2O$ powder were added to 2 mol L^{-1} nitric acid in a 1 : 1 stoichiometric ratio of Bi : (V + Mo) with vigorous stirring for 30min. Afterwards, 5 g urea was dissolved to the above-mentioned dispersion. The mixture was then constantly stirred at 90 °C for 24 h. The yielded Mo-doped $BiVO_4$ sediment was treated with filtering, washing, and drying at 100 °C for 12 h. The dopant concentration range [mol of Mo per mol of (V + Mo) in the precursor solution] investigated in this study is 1% - 5%.

1.3 Physicochemical characterization

The structure of Mo-doped $BiVO_4$ was investigated by a D/MAX 2500V/PC X-ray diffraction (Cu Kα radiation, λ = 0.15405 nm), operated at 40 kV and 30 mA. X-ray photoelectron spectroscopy (XPS) was carried out on an ESCALAB-MKII250 photoelectron spectrometer with Al Kα X-ray radiation as the X-ray source for excitation. High-resolution transmission electron microscope (HRTEM), selected area electron diffraction (SAED), element mapping and energy dispersive X-ray (EDX) spectrum were recorded with a TECNAI G2 microscope operating at 200 kV. Scanning electron microscope (SEM) images were taken on a FE-SEM XL30 ESEM-FEG at the accelerating voltage of 10.0 kV. Raman spectra were collected on a Renishaw Raman system model 2000 spectrometer using a 514 nm argon ion laser and calibrating referenced to the 520 cm^{-1} line of silicon. The UV-visible diffuse reflectance spectra (DSR, using $BaSO_4$ as the reference) were obtained from a Hitachi U-3900 spectrometer. Fluorescence emission spectra were performed on a Hitachi F-4600 fluorescence spectrophotometer. Except in an integrated device, all the electrochemical tests were measured at room temperature using a conventional three-electrode cell, comprising modified ITO or a glass carbon electrode (GCE, d = 3 mm) as working electrode, a platinum wire as the auxiliary electrode and a Ag/AgCl (3 mol L^{-1} KCl) as the reference electrode on a CHI920C electrochemical workstation. The PBS buffer as supporting electrolyte was bubbled with N_2 for at least 15 min and kept over a N_2 atmosphere during the experimental process. LED light (470 nm, Beijing Perfectlight Technology) was used as excitation source of the photoelectrochemical sensor. Electrochemical impedance spectroscopy (EIS) measurements were obtained using a Solartron 1255 B frequency response analyzer (Solartron Inc., UK) in a mixed solution of 1mmol L^{-1} $[Fe(CN)_6]^{3-/4-}$ and 0.1 mol L^{-1} KCl aqueous solution (amplitude 10 mV, 10^{-1} to 10^5 Hz). A Mott-Schottky plot was performed in 1 mol L^{-1} Na_2SO_4 with frequencies of 1000, 2000 and 5000 Hz.

1.4 Antioxidant Capacity (AC) Detection.

Fruits, and drinks were bought from the local supermarket. As for fruits, juice was collected from their flesh and then the supernatant liquid was further treated with centrifuge. As to teas, firstly, dry leaves (0.25 g) of each tea were suspended in 30 mL of boiling water. Then the tea was kept infusion for 30 min at room temperature. Finally the tea extracts were obtained via a normal

funnel. All the commercial drinks were used as received without further purification. The resultant samples were stored at a 4 ° C refrigerator.

The ITO electrode was cleaned with NaOH (1 mol L^{-1}) and H_2O_2 (30%), followed by sonicating in acetone and water, and then dried under ambient conditions. After that, 100 μL of bare $BiVO_4$ or Mo-doped $BiVO_4$ suspension (1 mg mL^{-1}) was cast onto the pretreated electrode within defined area. Through natural drying, the ITO electrode was further dried at 100 °C for 12 h to improve adhesion.

As illustrated in Figure 1a, the AC assay process on PEC sensor is clearly observed. In brief, once the buffer or sample was injected into the PEC cell, with light irritating from the back side of modified ITO electrode, the system instantly acquired electrical signal. Note that the final photocurrent was recorded and calculated as average value of three measurements at the same concentration. The PEC current was defined as following: $I = I_{sample} - I_{blank}$ (where I_{sample} is the photocurrent generated in the presence of sample, I_{blank} is the photocurrent generated in the absence of sample). Similarly, experimental procedures in our integrated device are nearly commensurate with the PEC sensor, which only has some little details different from it. Or to be more precise, a two-electrode cell is employed in this designed integrated device instead of the traditional three-electrode system. And the static cell used in PEC sensor is upgraded by the flow cell, where the injection rate is controlled with peristaltic pump at 10 r min^{-1}.

As control experiments, classical spectroscopic methods like DPPH and F-C approaches were also introduced to evaluate global AC in food. DPPH radical scavenging activity was determined as reported by Band-Williams *et al.* with minor modifications.[1] In short, 100 μL of Trolox standard solution or practical sample diluted solution was added into 900 μL of DPPH reagent (0.04 mg mL^{-1}, in absolute methanol). After reacting fifteen minutes, the change in absorbance at 514nm was immediately monitored by UV-vis spectroscopy. For F-C method, as described by Ainsworth,[2] briefly to the volume of 100 μL of GA solution or practical sample diluted solution, 200 μL (1/10 dilution) of F-C reagent was added. Then 800 μL of Na_2CO_3 (0.7 mol L^{-1}) was quickly mixed with the above solution. The mixture was incubated at 25 °C for 2h before reading the absorbance at 765 nm. All the results were replicated for three times. It is worth mentioning that DPPH assay is expressed as Trolox equivalents, while both F-C and PEC assays take GA as calibration standard.

2. Supplementary Figures and Table

A tiny distinction in width of band gap will entail a profound influence on photocatalytic activity. According to Fig. S1 below, energy band gap of $BiMo_{0.015}V_{0.0985}O_4$ is comparatively narrow among all the $BiMo_xV_{(1-x)}O_4$ samples.

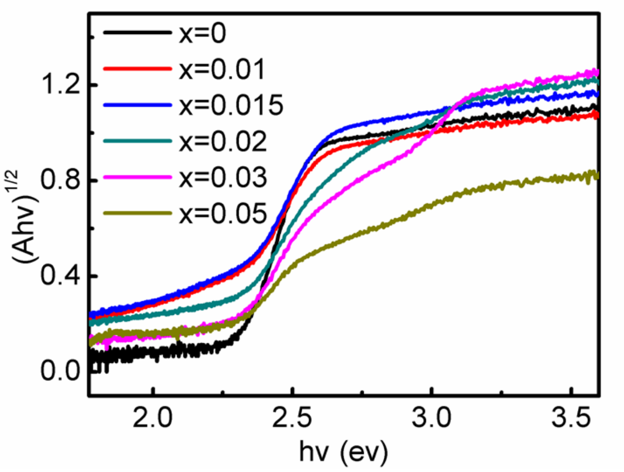

Fig. S1 The plot of transformed Kubelka-Munk function versus the energy of light for $BiMo_xV_{(1-x)}O_4$ samples.

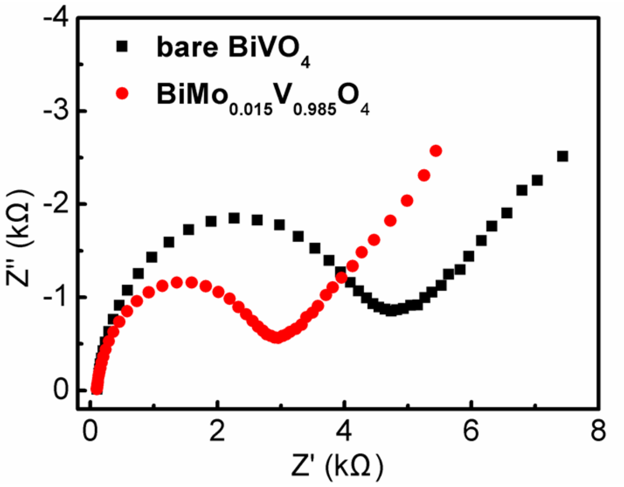

Fig. S2 EIS plots of bare $BiVO_4$ and $BiMo_{0.015}V_{0.985}O_4$ in mixed solution of 1mmol L^{-1} $[Fe(CN)_6]^{3-/4-}$ and 0.1 mol L^{-1} KCl solution at dark (amplitude 10mV, 10^{-1} to10^{-5} HZ).

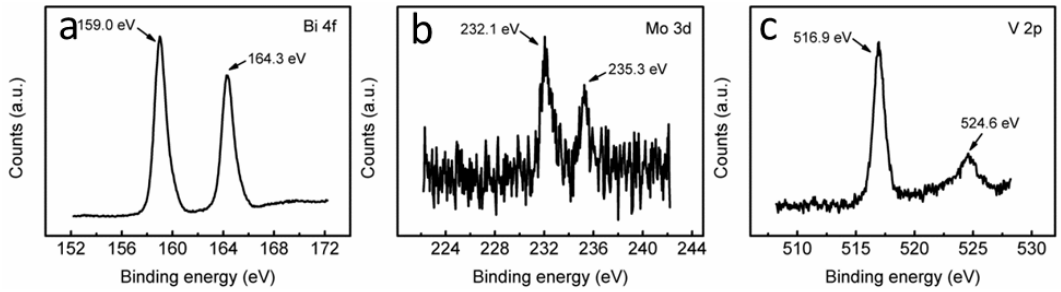

Fig. S3. (a) Bi 4f, (b) Mo 3d and (c) V 2p XPS spectrum of $BiMo_{0.015}V_{0.985}O_4$.

Current intensity or charge measurement is susceptible to change by the applied potential. Fig. S4 depicts that the photocurrent intensity gradually rose from -0.3 V to 0.3 V, followed by steady state from 0.3V to 0.6V. The positive potential is conducive to electron transfer through CB of semiconductor towards ITO electrode. The higher the potential applied, the greater the photocurrent expressed until the effect of electron concentration gradient is negligible. Since the current at 0 V accounted for 64% of 0.3 V, it is considerable for sensitivity performance. Meanwhile, the open circuit potential is also convenient for development of the integrated photoelectrochemical device. Thus at the comprehensive respect, 0 V was a suitable choice for PEC transducer.

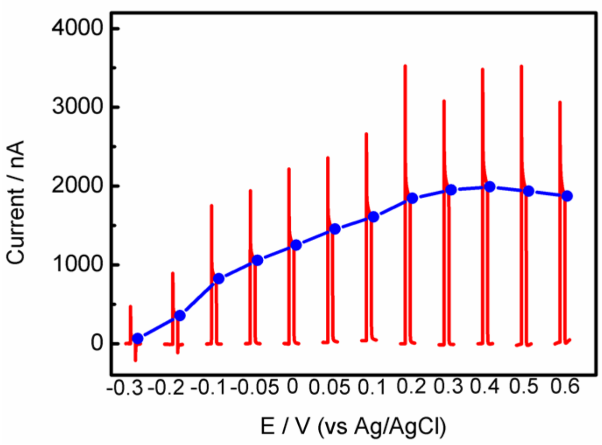

Fig. S4. Effects of the applied potential on photocurrent response of $BiMo_{0.015}V_{0.985}O_4$ modified ITO electrode in 0.1mol L^{-1} PBS (pH=7.4) containing 74.44 μmol L^{-1} GA under 470nm light excitation.

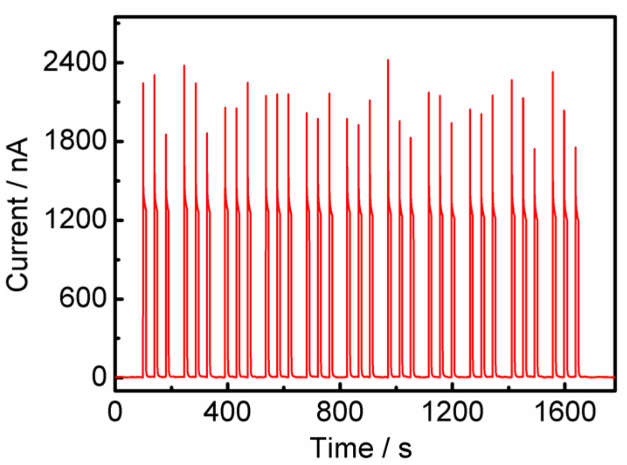

Fig. S5. Photocurrent response reproducibility for $BiMo_{0.015}V_{0.985}O_4$ modified ITO electrode in 0.1 mol L^{-1} PBS (pH=7.4) containing 74.44 μmol L^{-1} GA at 0 V under 470 nm light excitation.

Once hydroxyl radicals generate in the experiment, the special fluorescence signal will appear at 425 nm.[3] In reality, taken as a whole, any change was hardly discovered in 425 nm (Fig. S6). It could be inferred that antioxidants might react directly with the trapped holes rather than hydroxyl radicals.

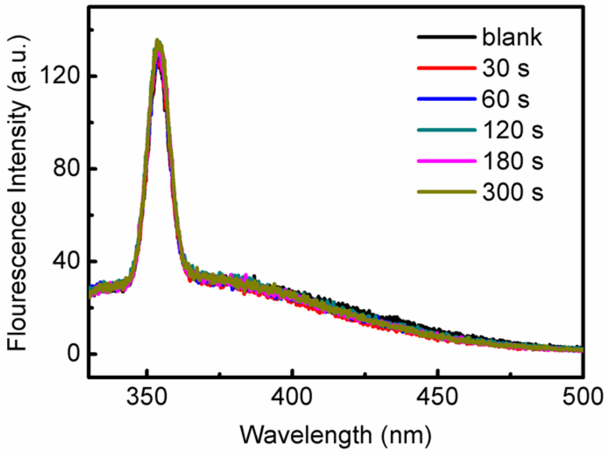

Fig. S6. Fluorescence emission of 0.5mmol L^{-1} terephthalic acid in 0.1 mol L^{-1} PBS (pH=7.4) with light of 0, 30, 60, 120, 180 and 300 s.

The flat band potential (E_{fb}) of semiconductor is closely related to the bottom of the conduction band (E_{cb}). As for many n-type semiconductors, E_{fb} is roughly at 0.1 V below the E_{cb}.[4] Hence, the position of conduction band is approximately equal to the flat band. Mott-Scottky analysis is a powerful tool to measure E_{fb}. Just as Fig. S7, the extrapolation Mott-Scottky plot intersect at -0.57 V (*vs* Ag/AgCl, namely, -0.363 V *vs* NHE), which sheds a brilliant light on E_{cb} value.

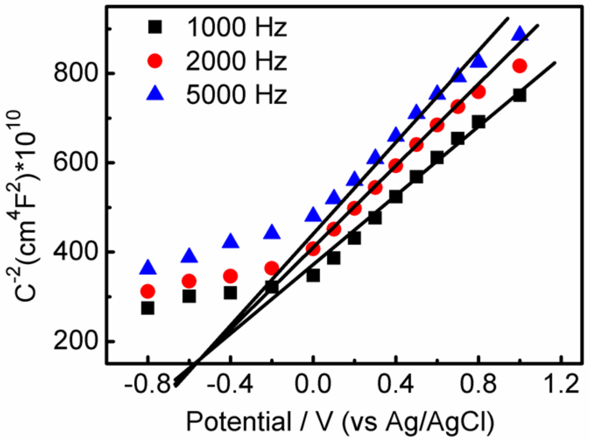

Fig. S7. Mott-Scottky plot of $BiMo_{0.015}V_{0.985}O_4$ modified ITO electrode in 0.1 mol L^{-1} PBS (pH=7.4) with frequencies of 1000, 2000 and 5000 Hz. The X-axis intercept is equal to the flat band potential E_{fb} which is the approximate value of CB.

To some degree, the redox potential is on behalf of reducing ability of an antioxidant. When no corresponding return cathodic peak is produced (as for some antioxidant molecules), the formal potential can be estimated by the potential halfway between $E_{p/2}$ and E_{pa} of the first peak.[5] (E_{pa} means the potential of anodic peak; $E_{p/2}$ means the half potential of anodic peak from the cathonic direction). The final results are listed in Fig. S8h.

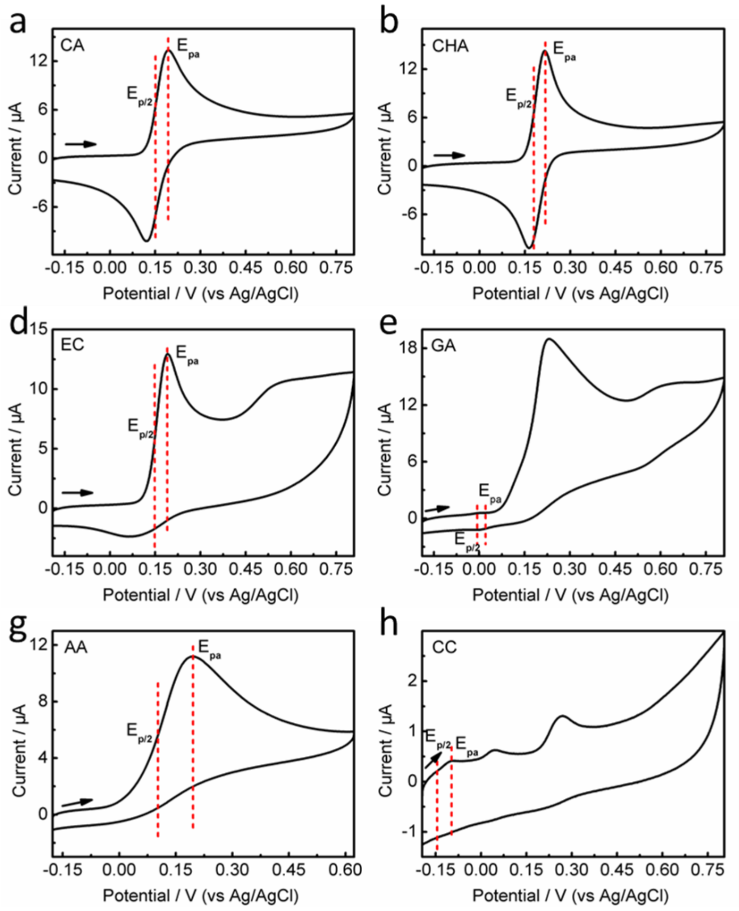

E in V	$(E_{pa}+E_{p/2})/2$ *vs* Ag/AgCl	$(E_{pa}+E_{p/2})/2$ *vs* NHE
CA	0.172	0.379
CHA	0.199	0.406
CT	0.192	0.399
EC	0.170	0.377
GA	0.007	0.214
MT	0.012	0.219
AA	0.149	0.356
CC	-0.121	0.086

Fig. S8. The Cyclic Voltammograms of 0.5 mmol L^{-1} (a) CA, (b) CHA, (c) CT, (d) EC, (e) GA, (f) MT, (g) AA and 0.1 mmol L^{-1} (h) CC. (i) Electrochemical characteristics of the first anodic peak of seven antioxidants using a 3-mm glassy carbon electrode at 0.1 V/s in 0.1 mol L^{-1} PBS (pH=7.4).

Table S1. Structure, linear equations, linear ranges and correlation coefficients (R^2) for eight antioxidants.

Antioxidants	Structure	Linear equation	Linearity range (μmol L^{-1})	R^2
**Gallic acid (GA)**	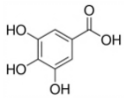	y = 7.812x + 49.022	6.23～304.81	0.996
**(-)-Epicatechin (EC)**	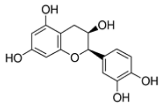	y = 2.245x - 11.909	12.46～815.90	0.999
**Chlorogenic acid (CHA)**	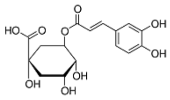	y = 1.313x - 32.269	24.87～1078.43	0.991
**(+)-Catechin hydrate (CT)**	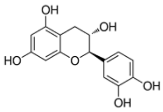	y = 3.335x - 90.921	37.22～633.19	0.998
**Caffeic acid (CA)**	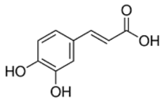	y = 2.270x - 139.066	61.73～833.33	0.995
**Ascorbic acid (AA)**	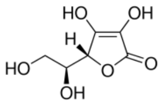	y = 0.431x - 4.098	12.46～327.10	0.987
**Cyanidin chloride (CC)**	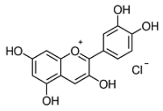	y = 2.315x - 10.705	4.95～215.91	0.977
**Myricetin (MT)**	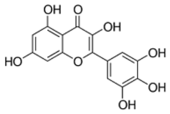	y = 5.942x + 64.978	12.46～433.79	0.957

Table S2. The different brand of commercial teas (T) and drinks (D) for AC monitoring.

T1	T2	T3	T4				
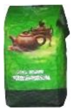	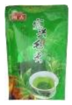	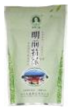	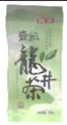				
**D1**	**D2**	**D3**	**D4**	**D5**	**D6**	**D7**	**D8**
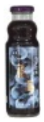	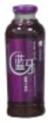	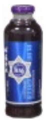	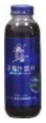	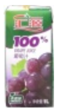	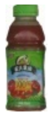	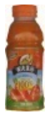	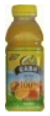
**D9**	**D10**	**D11**	**D12**	**D13**	**D14**	**D15**	**D16**
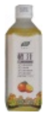	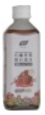	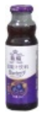	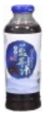	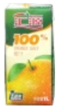	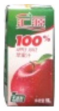	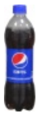	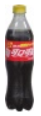

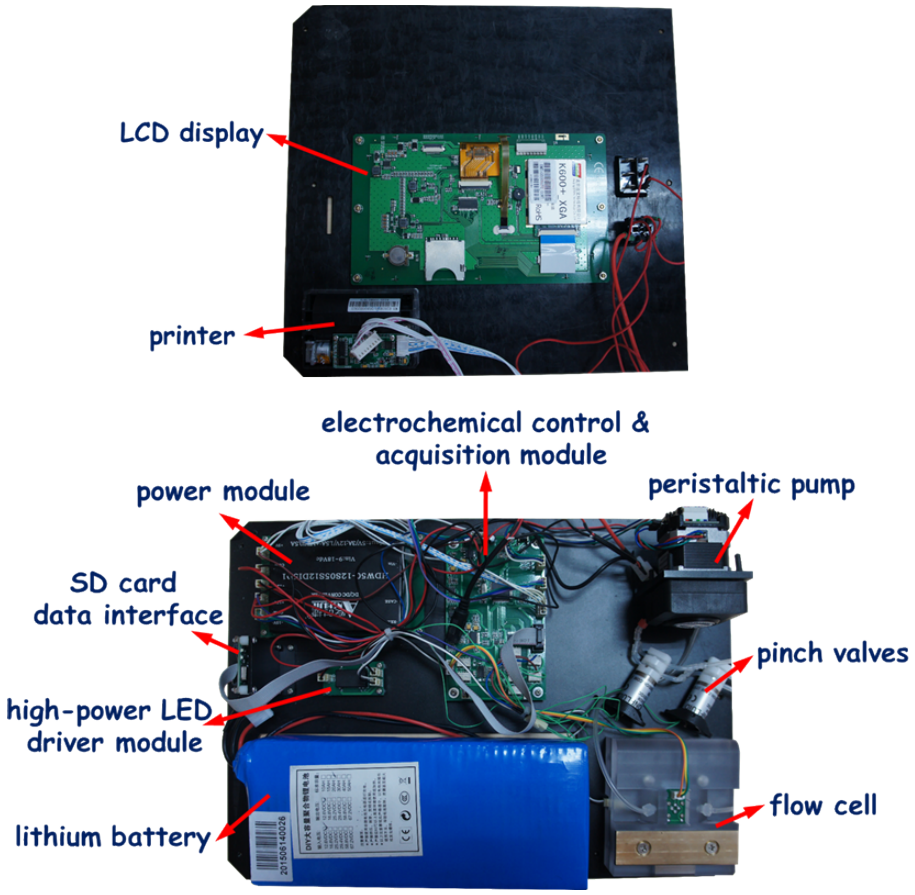

Fig. S9. The inner structure of the integrated device.

As shown in Fig. S10, it is observed that the amount of electrolytes hardly had any impact on the photochemical examinations towards the antioxidant (e.g. upon a 74.44 μmol L^{-1} GA solution).

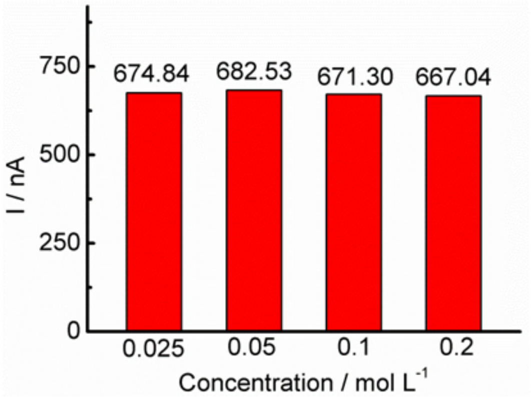

Fig. S10. Photocurrent responses of $BiMo_{0.015}V_{0.985}O_4$ modified ITO electrode in (a) 0.025 mol L^{-1}, (b) 0.05 mol L^{-1}, (c) 0.1 mol L^{-1}, and (d) 0.2 mol L^{-1} PBS solution upon 74.44 μmol L^{-1} GA at 0 V under 470nm light excitation. ($I = I_{sample} - I_{blank}$, where I_{sample} is the photocurrent generated in the presence of sample, I_{blank} is the photocurrent generated in the absence of sample).

As displayed in Fig. S11, after exposing 24 hours under natural light in room, the sensor still took up 91.23% of photocurrent response.

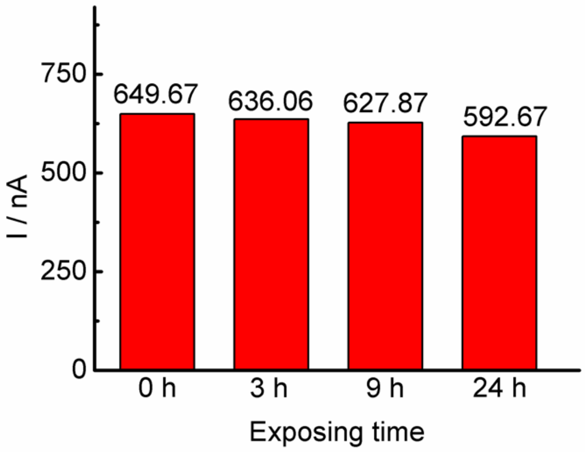

Fig. S11. Photoaging stability for $BiMo_{0.015}V_{0.985}O_4$ modified ITO electrode upon a 74.44 μmol L^{-1} GA while the electrode was stored in room natural light. ($I = I_{sample} - I_{blank}$, where I_{sample} is the photocurrent generated in the presence of sample, I_{blank} is the photocurrent generated in the absence of sample).

3. References

1 W. Brandwilliams, M. E. Cuvelier and C. Berset, *Food Sci. Technol.*, 1995, **28**, 25-30.

2 E. A. Ainsworth and K. M. Gillespie, *Nat. Protoc.* , 2007, **2**, 875-877.

3 J. F. Liu, C. Roussel, G. Lagger, P. Tacchini and H. H. Girault, *Anal. Chem.*, 2005, **77**, 7687-7694.

4 S. Sun, W. Wang, D. Li, L. Zhang and D. Jiang, *ACS Catalysis*, 2014, **4**, 3498-3503.

5 P. A. Kilmartin, H. L. Zou and A. L. Waterhouse, *J. Agr. Food Chem.*, 2001, **49**, 1957-1965.
